# Students' boredom in English language classes: Voices from Saudi Arabia

**DOI:** 10.3389/fpsyg.2023.1108372

**Published:** 2023-03-07

**Authors:** Abdul Aziz Mohamed Mohamed Ali El Deen

**Affiliations:** Department of English Language and Literature, College of Languages and Translation, Imam Mohammad Ibn Saud Islamic University (IMSIU), Riyadh, Saudi Arabia

**Keywords:** classroom boredom, academic boredom, Saudi EFL learners, English classes, blended learning

## Abstract

Although academic research on English-as-a-foreign-language (EFL) emotion has recently increased, there is a paucity of studies related to boredom in the Saudi context. To address this issue, the present study aimed to identify the causes of students' boredom in English classes in the Saudi context and the fluctuations of boredom experienced by students while taking language skill courses. Utilizing a mixed-methods design, the study drew upon a questionnaire and semi-structured interviews. The questionnaire was completed by 356 participants from different EFL programs in Saudi universities, and the interviews were conducted with 20 students. The results of this study provided a detailed account of the causes of boredom in the Saudi EFL environment. Specifically, an exploratory factor analysis revealed the following seven factors that account for boredom: demotivation, low language learning ability, type of skills-based courses and over-challenging tasks, unfamiliar instructional techniques, teacher feedback areas, under-challenging tasks, and classroom mode, permanent correction, and redundancy. The study also highlighted boredom fluctuations in courses based on language skills *via* the use of descriptive statistics (means, standard deviations, and percentages). Compared to other courses/lessons, the students of EFL programs were found to have the highest boredom levels in grammar and writing. The study ends with a discussion on the implications of the results and provides suggestions for future research.

## 1. Introduction

Learning a second or a foreign language is accompanied by both positive and negative feelings. Feelings can be either positive such as joy and interest, or negative such as apprehension, shame, and anxiety. Since students' negative feelings are barriers to their language learning, the effect of these negative feelings on language learning needs to be intensely researched (Teimouri, 2018). Despite its complicated, painful effects on language learning, classroom boredom has been an under-researched area (Li and Dewaele, 2020; Mercer and Dornyei, 2020; Pawlak et al., 2020a,b; Derakhshan et al., 2021; Li, 2021; Nakamura et al., 2021). Boredom can also be viewed as an affective domain, including multi-sub constructs such as anger, demotivation, depression, frustration, grit, and other aspects. One of the causes of the scarcity of research on boredom is that boredom is sometimes difficult to recognize in others (Nett et al., 2010).

Boredom in a second/foreign language classroom is poorly perceived, and research on this topic is still scant. In the foreign language education area, the construct of language classroom boredom was introduced in applied linguistics research on by Chapman (2013). An important development in the research on language learning affect is the realization of the important role of emotions, not just cognition in teaching and learning to teach (Gkonou and Miller, 2021). Though boredom is perhaps the most common emotion in foreign language classes, less attention has been given to it as compared to other affective variables such as anxiety, demotivation, and depression (Pawlak et al., 2020c). Specifically, there is a need for conducting research on boredom in online language learning environments. Zembylas (2008) states that the research on affect in virtual learning settings can only be fostered through a deep investigation of positive and negative emotions and feelings. Many studies have been conducted on the nature of boredom in majors like psychology, education, and educational psychology (Mora, 2011; Sharp et al., 2017; Van Tilburg and Igou, 2017); however, research concerned with boredom in foreign language settings is still scarce. The number of recent studies dealing with boredom in EFL contexts is still low (Kruk and Zawodniak, 2018; Shehzad et al., 2020). According to Tze et al. (2016), more attention should be given to conducting research on boredom in language learning since it is one of the most important affective variables in academic environments. Macklem (2015) suggests that boredom has been neglected by instructors who normally attribute it to learners' anxiety, frustration, or passive personality factors. In this regard, it was noted that there has been emphasis of research on the impeding impact of boredom on the learning process. On the other hand, research shows that disengagement is an essential dimension in classroom boredom (e.g., Skinner et al., 2009; Veiga et al., 2014; Chen and Kent, 2019).

Arguably, language classroom boredom is influenced by contextual factors, and these factors vary from one learning environment to another. While previous studies tackled the causes of classroom boredom in many international EFL settings, the subject of classroom boredom has not received due attention in Saudi Arabia. Given this contextual gap, the present study attempted to examine the causes of boredom in English classes in the Saudi context and how these causes vary in language skill courses.

## 2. The nature of classroom boredom

Classroom boredom is a multi-dimensional concept. It can be defined from different angles, and its nature is close to that of a school setting. In spite of the surge in the spread of boredom construct in the academic affective domain, it has been an unmapped region for second/foreign language researchers. According to many researchers (e.g., Larson and Richards, 1991; Preckel et al., 2010), boredom can be termed as a unique trait of low-ability and low-achieving learners who are unable to confirm what obstacles stand in their way and how failure could be avoided. However, this negative emotion might also represent an obstacle, even to talented students who do not encounter a challenging task; this causes them to miss classes or express less enthusiasm. Pertinent literature indicated that the causes of boredom are numerous and that they can be related to the nature of the task (Graesser and D'Mello, 2012), lack of attention and interest, or demotivation (Graesser et al., 2014). Disengagement can be defined as a lack of enthusiasm, distraction instead of effort, and disillusionment, which made the learners withdraw from different situations where others can be involved in. It is connected with impulsivity, unwillingness, and demotivation. Therefore, disengagement is an obstacle to language learning and a crucial passive aspect of boredom (Dornyei, 2019).

As regards the type of classroom boredom, Goetz et al. (2014) categorize boredom into different types. The first type is indifferent boredom, which indicates a pleasant state of calmness and withdrawal. The second type, which is termed calibrating boredom, is related to a moderately unpleasant state of dispersed thought. The third type represents the positive side, as it can urge creative learners to search for solutions. The fourth type represents unpleasant emotion because learners prefer to avoid it. The last and the most negative type is pathetic boredom with which learners become frustrated. Accordingly, it can be concluded that the construct of boredom does not include negative traits only, but it also has some positive dimensions that could foster the learning process.

Some theories and models explicate the nature of boredom. In their under-stimulation model, Larson and Richards (1991) view that boredom is the result of the absence of new stimuli and learning environments. In this respect, the emphasis is given to repetition, memorizing, and rote learning rather than problem-solving activities or novelty. Other researchers (Hill and Perkins, 1985; Pekrun et al., 2010) ascribe boredom to poorly designed tasks by relying on a model, namely, the forced-effort model. This model attributes boredom to the characteristics of learners who do not take responsibility for their own learning. Such learners are being induced to carry out repetitive, monotonous tasks.

Other psychological theories also highlight some dimensions of boredom. For example, according to the attentional theory, the main cause of boredom is inattention or failure to remain focused on uninteresting and unengaging tasks (Harris, 2000; LePera, 2011). However, the control-value theory of achievement emotions states that learners lose interest in the task at hand when they do not find it meets their perceptions (Pekrun, 2006). Similarly, the cognitive–motivational model of emotion effects elucidates the impact of boredom and academic emotion on students' achievement. According to this model, engagement and boredom occur as a result of over-challenging or under-challenging tasks (Davies and Fortney, 2012). As regards the dimensional model and boredom, Pekrun et al. (2010) emphasize on the limelight side of the emotion and its complicated aspects. Accordingly, academic boredom is a multi-dimensional psychological phenomenon which is of different types and which interacts with many variables.

## 3. Causes of foreign language classroom boredom

Practically speaking, there are many teaching practices that could cause second language (L2) learners to experience boredom. For example, some learners occasionally glance at their mobile phones or raise their hands to go out as an excuse to escape from the class. Research indicates that classroom boredom has a deleterious influence on the learning process (Tze et al., 2016). Some studies found that boredom occurs as a result of different factors such as the absence of new stimuli, the nature of rote learning, and poorly designed and repetitive tasks e.g., (Hill and Perkins, 1985; Larson and Richards, 1991; Pekrun et al., 2010; Davies and Fortney, 2012). In a similar vein, boredom could be evoked by over-challenging or under-challenging tasks, where learners experience either a deficit or a surplus of mental energy units. According to Derakhshan et al. (2021), in online classes, boredom can also be felt as a result of teacher-related, task-related, and student-related factors. Derakhshan found that teacher-related factors—such as instructional practices and personality—represent the most common factor causing feelings of boredom.

Some studies investigated the predictors of boredom in traditional and virtual EFL classes. For example, Chapman's (2013) study shows that the major factors of boredom include under-challenging activities, course book-based activities, and peer students who are not involved in the courses. In a mixed-methods study, Pawlak et al. (2020b) found fluctuations in English majors' boredom experiences as in the Polish environment. Their study findings indicate that speaking activities showed a low level of boredom compared with grammar, writing, and reading activities. In Zawodniak et al.'s (2021) study, they found a set of factors accounting for English majors' boredom. These factors were categorized through a qualitative analysis as follows: tasks related to language, the instructor, classroom organization, class courses, and other miscellaneous factors. Though 115 participants took part in Zawodniak et al.'s research, their study did not provide a detailed analysis of the variance in students' boredom in skills-based courses.

There are some research gaps that are yet to be addressed in the research on language classroom boredom. First, there is a need for examining the construct of boredom to minimize its detrimental effects on language learning (e.g., Tze et al., 2016; Kruk and Zawodniak, 2018; Shehzad et al., 2020). Second, there is also a need for investigating the extent to which boredom varies in skills-language courses. Third, language classroom boredom is still under-explored in some contexts. For example, it has been recently investigated in the Polish environment (Pawlak et al., 2020a) and also in some Asian settings (e.g., Li and Dewaele, 2020; Derakhshan et al., 2021; Li, 2021; Li et al., 2021; Nakamura et al., 2021). To the researcher's best knowledge, no studies have yet been conducted to investigate the nature and identify causes of boredom in the Arab EFL context, in general, and in Saudi EFL classes, in particular. The only two previous relevant studies in the Saudi context solely focused on the relationship between boredom, reading, and listening from a narrow angle (Shehzad et al., 2020, 2021). Fourth, the main concern of numerous studies on boredom has been focused on regular face-to-face classes. Thus, there is scant literature on boredom in online or blended English classes, particularly in view of the COVID-19 pandemic (Pawlak et al., 2022). Finally, a methodological gap lies in the need for designing an assessment tool that measures the numerous potential causes of boredom in various English classes. One merit of using the questionnaire is to access numerous participants and investigate the potential sources of boredom (Dumančić, 2018; Kruk and Zawodniak, 2018; Pawlak et al., 2020a; Nakamura et al., 2021; Zawodniak et al., 2021).

Taking these research gaps into account, the present study investigated the causes of boredom among Saudi university EFL students. In this study, the emphasis is placed on boredom in skills-based courses or English components. The significance of the present study lies in conducting research on boredom in foreign language blended classes. The originality of the study also lies in the questionnaire used in it. All these aspects could lead to the provision of important implications for English language education practices in Saudi Arabia.

## 4. The present study

### 4.1. Context and research questions

This study was conducted in a Saudi university context, in which the participant students were studying their English language courses in a blended learning mode. These students were completing a foundation year program. Due to COVID-19 protection measures, each class was divided into two groups: the first group had to attend face-to-face classes in 1 week and the second group had to attend the same face-to-face lessons in the following week. In the third week, the two groups had online classes in all language courses. Accordingly, the students had a mixture of face-to-face lessons and online classes during one academic term. As implied, the present study is guided by the following two research questions:

1. What are the causes of students' boredom in blended English classes in Saudi universities?
2. How do students' experiences of boredom fluctuate in different types of language-learning activities?

To answer the two research questions, the present study adopted a mixed-methods design by using a questionnaire and semi-structured interviews.

### 4.2. Participants

The participants of the present study comprised 356 questionnaire respondents and 20 interviewees. They were completing the foundation year program at the following four Saudi universities: Imam Mohammad Ibn Saud Islamic University, Majmaah University, Shaqra University, and Princess Nourah Bint Abdulrahman University. Of these 356 participants, 279 were men and 77 were women. As for the interviewees, they were all male students. Depending on the maximum variation sampling technique, 356 participants were selected to take part in the current study. The foundation year programs that these students were attending included different intensive courses covering all language areas: listening, speaking, reading, writing, vocabulary, and grammar. The very vast majority of the students studying in these programs are Saudis. The author obtained institutional approval for collecting data before conducting the study. Additionally, informed consent was obtained from the participants after introducing the study to them, explaining its purposes, and confirming the confidentiality of their identities. All participants agreed to take part in the study voluntarily.

### 4.3. Data sources

The two instruments of this study—i.e., the questionnaire and semi-structured interviews—were developed in light of the purpose of the study and also guided by a thorough review of related literature written on classroom boredom in educational psychology and language learning (e.g., Pekrun et al., 2010; Dumančić, 2018; Pawlak et al., 2020c; Nakamura et al., 2021; Kruck et al., 2022). The questionnaire starts with an introduction and a section on demographic information, which was used to collect data about the participants' age, gender, and university details. It includes 35 items that assess the causes of students' boredom. These 35 items are given in four sections that assess the symptoms of classroom disengagement and monotony (items 1–8), task-related boredom (items 9–23), teacher-related boredom (items 24–30), and language learning ability-related boredom (items 31–35). A 5-point Likert scale of 1 to 5 was used for rating these items (5 = always, 4 = often, 3 = occasionally, 2 = seldom, and 1= never). In addition to these statements, the questionnaire includes four open-ended questions; one question is given under each section (see the Questionnaire in Appendix A). The questionnaire was translated into Arabic to facilitate the respondents' understanding of its items, given that Arabic is their mother tongue. To verify the validity of the questionnaire, four expert researchers read its items to evaluate their phrasing and suitability for the purpose of the study and the boredom dimension it assesses. In light of their views, a few modifications were made to the phrasing of some statements. As regards the reliability of the questionnaire, it was found that it has a Cronbach's alpha coefficient of 0.915, which is a high-reliability level.

As regards the semi-structured interviews, they were used as a way of data triangulation to gather further data about the students' inner feelings and experiences of boredom in English classes. The five guiding questions of the interviews focused on the students' feelings of boredom and their symptoms in English classes, and the causes of their boredom and variance in it. The interview questions were written in Arabic to facilitate communication. It was also planned to raise follow-up interview questions to each interviewed student depending on their answers to the main guiding questions.

### 4.4. Data collection and analysis

The data were collected over a period of 2 months. The Arabic version of the questionnaire was written using Google Forms, and then the students were invited to complete it electronically. The link of the questionnaire page was circulated by the author and his workplace colleagues to intact classes of the target student population in the four universities mentioned. Responses to the questionnaire were received during the 2 months, finally reaching 356. However, the interviews were conducted within a duration of 2 weeks with 20 participants who were studying at the author's workplace. These students were randomly invited to take part in the study. All the interviews were conducted on a face-to-face basis. Each student was interviewed individually for about 20–25 min, and all interviews were audio-recorded. After the collection of the interview data, they were transcribed and translated from Arabic into English.

The quantitative data of the questionnaire were analyzed by calculating the means, standard deviations, and percentages of each item and using the exploratory factor analysis. The principal component analysis using rotation sums of squared loadings procedure was conducted on the 35 items of the questionnaire. Additionally, the students' answers to the open-ended questions of the questionnaire were analyzed qualitatively. Similarly, the author read the transcribed interview data numerous times and analyzed them qualitatively. To validate the data analysis of the initial interview, an expert researcher read and analyzed part of it, and there was a high agreement between him and the author on the analysis units. The emerging themes in the whole interview data were identified and categorized.

## 5. Results of the data analysis

In this section, the author provides the results of the data analysis in accordance with the two research questions. Subsection 5.1 provides the data related to the first research question and subsection 5.2 provides the data related to the second research question.

### 5.1. Causes of students' boredom in blended versus face-to-face English classes

The quantitative analysis of the students' responses to the questionnaire showed important dimensions about the causes of students' boredom in English classes. The EFA revealed a detailed framework of seven main factors accounting for the students' boredom. Table 1 shows the results of the exploratory factor analysis and the loadings of the questionnaire items. As the table shows, the first factor or cause of boredom (i.e., demotivation) has eight items (3, 6, 4, 5, 8, 13, 1, and 2) with high loadings (α = 0.867). These eight items indicate cases of enthusiasm, distraction, lethargy, grit, and frustration. The second factor or cause encompasses five items (items 33, 35, 31, 32, and 7) with high loadings as well (α= 0.849). These items concern students' perceived academic weaknesses and low abilities in certain language areas and lack of concentration (e.g., item 7). Taking into consideration the nature of aspects these items assess, it can be concluded that these items can be labeled as defects in language learning ability. The third factor includes eight items (item, 21, 20, 17, 22, 23, 19, 18, and 10) with similar loadings (α = 0.825). Seven items in this factor are related to the nature of skills-based courses, and their degrees vary according to the order given in Table 2 and one item closely related to the difficulty of learning activities (item 10). Overall, the eight items can be categorized as related to skills-based courses. Three items (14, 15, and 16) represent the fourth factor, which concerns learning through unfamiliar instructional techniques. The item loadings of this fourth dimension are higher than the other factors (α = 0.922). One item is related to performing learning activities in a self-learning method (item 14), and the other two items are related to experiencing different learning activities, either through pair or through group activities (items 15 and 16). The fifth dimension has six items (29, 30, 25, 26, 24, and 27) with 0.789 item loadings. Most of these items are strongly associated with teachers' feedback types. Only item 30 is related to the humanistic atmosphere. These items reflect the role played by teacher feedback in shaping EFL students' classroom boredom or motivation.

**Table 1:** Results of the exploratory factor analysis and the loadings of the questionnaire items.

**1st factor: Demotivation (α = 0.867)**	
Item 3- I do not feel like doing anything in English classes.	0.736
Item 6- I just sit around doing nothing in English classes.	0.728
Item 4- It is not easy for me to concentrate in English classes.	0.694
Item 5- During English classes, I often think about unrelated things.	0.692
Item 8- I do not feel entertained or excited in English classes.	0.680
Item 13- I feel bored in English classes when doing face-to-face learning activities.	0.581
Item 1- Time passes slowly in English classes.	0.545
Item 2- It takes me more time to get engaged in English classes than other students around me.	0.483
**2nd factor: Language learning ability (**α = **0.849)**	
Item 33- I am not efficient in the language area covered in the learning activity.	0.806
Item 35- I feel my English level is much lower than my peers.	0.786
Item 31- I feel bored in English classes because I have a limited English language ability.	0.781
Item 32- I feel bored in English classes because I cannot see any progress in my English language ability.	0.668
Item 7- I often find myself at a loose end in English classes.	0.528
**3rd factor: Skills-based courses and over-challenging tasks (**α = **0.825)**	
Item 21- I feel bored in English classes when doing writing activities.	0.683
Item 20- I feel bored in English classes when doing pronunciation activities.	0.619
Item 17- I feel bored in English classes when doing grammar activities.	0.619
Item 22- I feel bored in English classes when doing vocabulary activities.	0.567
Item 23- I feel bored in English classes when doing reading activities.	0.543
Item 19- I feel bored in English classes when doing speaking activities.	0.521
Item 18- I feel bored in English classes when doing listening activities.	0.474
Item 10- I feel bored in English classes when the learning activities are very difficult.	0.375
**4th factor: Learning through unfamiliar instructional techniques (**α = **0.922)**	
Item 14- I feel bored in English classes when doing learning activities on my own.	0.917
Item 15- I feel bored in English classes when doing group learning activities.	0.862
Item 16- I feel bored in English classes when doing pair activities.	0.860
**5th factor: Teacher feedback areas (**α = **0.789)**	
Item 29- I feel bored in English classes when the teacher does not correct my mistakes.	0.747
Item 30- I feel bored in English classes when the teacher is not friendly.	0.726
Item 25- I feel bored in English classes when the teacher has excessive control over the class.	0.678
Item 26- I feel bored in English classes when the teacher does not engage me in learning activities.	0.586
Item 24- I feel bored in English classes when the teacher has unchanging instructional routines.	0.453
Item 27- I feel bored in English classes when the teacher overloads me with language information.	0.433
**6th factor: Under-challenging tasks (**α = **0.745)**	
Item 9- I feel bored in English classes when learning activities are easy.	0.724
Item 34- I feel bored in English classes when I feel my English level is much higher than my peers.	0.613
**7th factor: Classroom mode, permanent correction, and redundancy (**α = **0.723)**	
Item 12-I feel bored in English classes during online learning activities.	0.575
Item 28- I feel bored in English classes when the teacher corrects my mistakes.	0.440
Item 11- I feel bored in English classes when doing similar learning activities.	0.369

**Table 2:** The means, standard deviation, and percentages of the participants' responses to the questionnaire items related to boredom in skills-based courses.

**Item**	**Mean**	**SD**	**Percentage**
17- I feel bored in English classes when doing grammar activities	2.77	1.28	55.40%
21- I feel bored in English classes when doing writing activities	2.72	1.3	54.40%
18- I feel bored in English classes when doing listening activities	2.33	1.22	46.60%
19- I feel bored in English classes when doing speaking activities	2.23	1.24	44.60%
20- I feel bored in English classes when doing pronunciation activities	2.19	1.17	43.80%
23- I feel bored in English classes when doing reading activities	2.13	1.25	42.60%
22- I feel bored in English classes when doing vocabulary activities	2.11	1.12	42.20%

The qualitative data provided support for the aforementioned quantitative analysis related to dimension 5. For example, a questionnaire respondent provides the following answer that supports item 29:

*I feel bored when the teacher does not correct my mistakes or ignores me, moreover, when there is also a redundancy of exercises. (questionnaire respondent 136)*

Another questionnaire respondent's answer confirms the loading of item 24. He states that:

*I feel bored when the teacher does not make any variety in his teaching methods. (questionnaire respondent 23)*

Similarly, the following answer reflects both items 30 and 27:

*I get bored if the instructor is strict and provides me with a large number of assignments. (questionnaire respondent 139)*

Finally, some questionnaire answers concur with item 27. For example:

*I feel bored when the instructor selects difficult words during explanation instead of simple ones. (questionnaire respondent 174)*

With regard to the sixth dimension, it has item loadings of 0.745. It includes two items (9 and 34). One item is related to the ease of the learning activities (item 9) and the other one is associated with the high academic abilities of the learner (item 34). The two items relate to the boredom feelings resulting from under-challenging activities. Finally, the seventh dimension has item loadings of 0.723. This dimension includes three items (12, 28, and 11). One of these items reflects the influence of classroom mode (item 12). The other two items (items 28 and 11) relate to permanent correction and similar learning activities. The items in this seventh dimension concern classroom mode, teacher correction, and redundancy.

### 5.2. Changes in the degree of boredom in skills-based courses

In answering the second research question, the author drew upon combining the quantitative and qualitative data. The qualitative data here include the answers to both the questionnaire's open-ended questions and semi-structured interviews. As regards the fluctuation of boredom in English classes, Table 2 shows the descriptive data of the participants' responses to the questionnaire items related to boredom in skills-based courses. As noted, differences are noticed in the students' reported levels of boredom in the English courses they attend. The data show that item (17), which is related to performing grammar activities, is associated with the highest mean of feelings of boredom. The questionnaire respondents' answers support this higher boredom level in grammar lessons, for example:

*I feel bored when studying some courses, particularly grammar, because teachers don't vary their techniques and they don't lighten-up the classroom atmosphere. (questionnaire respondent 74)*

*I get bored in grammar classes because of repetition of the lessons from the first year in primary stage till now. (questionnaire respondent 134)*

*What makes me feel bored is the repetition of the lessons from intermediate stage until secondary stage. (questionnaire respondent 136)*

Similarly, the semi-structured interview data revealed similar issues related to the boredom feelings the students experience in grammar lessons, for example:

*I get bored in grammar classes, as the teacher only reads. He doesn't ask at all. Moreover, he does not use PowerPoint presentations in explaining grammatical points. (interviewee 5)*

*I get bored in grammar classes. They include the same activities, and they are full of redundancy. (interviewee 8)*

The data also show that writing represents the second highest boredom level mean (mean of item 21= 2.72). This boredom level mean could be due to the nature of difficulties that the students encounter while writing in English. The qualitative data provide explanations for this difficulty in writing in English. For example, one questionnaire respondent attributed this difficulty to his negative attitude toward writing:

*I get bored as I don't like the writing course. (questionnaire respondent 179)*

The interviews also revealed other causes for students' boredom in writing lessons, such as too much time needed for writing tasks, lack of support, and inability to generate ideas. For example:

*I get bored in writing classes when there is too much writing and when there is no collaborative work among us … In addition, I don't find effective techniques in learning writing. (interviewee 6)*

*To be honest, I hate writing paragraphs and I feel a sort of difficulty when the teacher asks me to write. I don't know how I can elaborate my ideas. (interviewee 10)*

Listening is the language area with the third highest boredom mean (mean of item 18 = 2.33). The participants' answers show that the boredom in listening in lessons can be attributed to the difficulties they have in understanding and to the nature of listening comprehension questions, for example:

*I get bored in the listening course as I have difficulties while answering questions related to listening activities. (questionnaire respondent 174)*

*I get bored in listening activities when the excerpt is too long and I am not capable of understanding the nature of questions. (interviewee 11)*

As regards speaking activities, it is the language area with the fourth highest boredom mean (mean of item 19 = 2.23). Pronunciation activities have a close mean (2.19). Some questionnaire respondents and interviewees explained that this value is due to the difficulty in studying pronunciation activities, and their low rating of the teachers' pronunciation performance. This can be noted in the following answers:

*I get bored in pronunciation classes as I find difficulty in studying pronunciation activities. (questionnaire respondent 198)*

*I get bored when the instructor mispronounces certain words. (questionnaire respondent 222)*

*I get bored in speaking activities when the teacher pronounces difficult words….. I remember I get confused when the teacher elucidates linking sounds, particularly, /j/ and /w/ sounds. Yet, sometimes, I feel enthusiasm when I learn new rules in phonetics. (interviewee 12)*

The students' questionnaire responses indicate that both vocabulary and reading have the lowest boredom levels (means of items 22 and 23 = 2.11 and 2.13, respectively). This can be interpreted by the fact that the two areas are interrelated, given that students study or come across new vocabulary in reading lessons more than in any language area. In addition, the qualitative data show that the students could experience some boredom in reading classes due to factors such as the text length and the teaching instructional method used:

*I feel bored in reading classes when the text is too long and I am not reading the text by myself. (questionnaire respondent 93)*

*In the first semester, I didn't get bored in the reading course. Reading course was the best course for me. However, in the second semester, I hate the reading course and it has become the worst one for me … because of the change in the instructor and teaching method. (interviewee 13)*.

## 6. Discussion

This study examined the causes of boredom in blended Saudi university EFL classes and the fluctuations of the students' boredom experiences during the English classes. The causes of boredom, as revealed by the present study, concur with Mercer and Dornyei's (2020) view that it is hard to separate the construct of boredom from other constructs such as demotivation, apprehension, reticence, and burnout. The results of the study also emphasize the importance of varying learning activities. In their simulation model, Larson and Richards (1991) postulate that boredom stems from neglecting novel stimuli and problem-posing learning environments and depends on memorization. This model interpreted the loadings of items 3, 6, 4, 5, 8, 13, 1, and 2. With regard to the main factors causing boredom in the Saudi university EFL context, the main factor relates to the students' low abilities in academic achievement. This is clear from the students' responses to some questionnaire items (33, 35, 31, and 32). In this regard, the results of the present study are congruent with those of emotion theory (Eastwood et al., 2007; Zawodniak et al., 2021), which suggests that the main cause of students' boredom is the difficulty in realizing and sharing their emotions with others.

This study also emphasizes the importance of teacher feedback as a potential factor of students' boredom. Therefore, this finding goes in line with Derakhshan et al.'s (2021) findings that the most influential types of boredom causes are those related to the teacher. In this study, Saudi university EFL students ascribe their boredom in English classes to the feedback types provided by their teachers. In addition, the present results go in line with the Menton theory of engagement and boredom (Davies and Fortney, 2012), which suggests that under-challenging tasks can cause students to experience boredom. In other words, if students do not have adequate mental imagery units, they will feel bored in their learning environment (Davies and Fortney, 2012).

As regards the fluctuation of learners' experiences of boredom in skills-based courses, the present study indicates that students have the highest boredom levels in grammar and writing lessons. The qualitative data particularly showed that participants reported boredom experiences in grammar classes. Consequently, these results are consistent with those of Jean and Simard's (2011) study. In a similar vein, the quantitative and the qualitative analyses confirmed the high difficulty and boredom levels the Saudi EFL learners encounter during writing courses. Thus, the current study supports Al-Fadda's (2012) research findings about Saudi EFL students' poor performance in English writing. Similarly, the study showed that the students' boredom in listening activities stems sometimes from their difficulties in listening activities. As for speaking and pronunciation activities, it is apparent through interviews that the students have a lower boredom level in speaking activities, but some boredom levels in pronunciation activities could be ascribed to the students' deficiencies in dealing with them. Although reading activities are rated by the students as less boring, the qualitative data indicate that some students may experience boredom while reading due to teaching methods or text features. It can be concluded that the qualitative data can reveal deeper insights into students' emotional experiences.

The main contribution of this study is its investigation of boredom fluctuations in skills-based courses. Shehzad et al. (2021) state that, although research has given due attention to boredom in different contexts, fewer studies have looked at the association of boredom with language areas. This study also emphasizes the role of the learning mode in the students' boredom. Some participants reported that working individually is a boring activity, while others deemed pair or group work very boring. Finally, the variance of boredom in the seventh dimension indicates that a high rate of boredom is experienced by students in Saudi university's English blended classes.

## 7. Conclusions and suggestions for further research

Though boredom has been a dominant research area in educational sciences, it is still under-explored in language education research (Lee and Lee, 2020; Yuan, 2020). The current study is an endeavor to understand Saudi EFL learners' practices of boredom in blended classes. Exploratory factor analysis showed that Saudi university EFL students' boredom could stem from the following seven factors: demotivation, low language learning ability, type of skills-based courses and over-challenging tasks, unfamiliar instructional techniques, teacher feedback areas, under-challenging tasks, and classroom mode, permanent correction, and redundancy. Moreover, the students were found to have the highest boredom levels in grammar, writing, and listening—respectively—than in the other language areas. The qualitative data obtained from both the questionnaire and semi-structured interviews confirmed the quantitative results. Overall, the study shows a moderate level of boredom in all dimensions. However, the nature of blended classes contributed to mitigate the level of boredom in an indirect way.

As regards the neglected negative construct of boredom in an EFL/ESL environment, and in the Saudi EFL context in particular, the following issues could be reconsidered as ideas for future research. One of these points is identifying coping strategies to mitigate or relieve boredom of Saudi EFL learners and instructors. Furthermore, future research could also investigate the relationship between boredom and learning styles in the Saudi university EFL environment. Future studies could also look at the impact of electronic assessment on minimizing boredom at the university level. The relationship between language skills, particularly writing and speaking, and boredom, should be reconsidered. Finally, further research is required to investigate boredom coping strategies in Saudi EFL classes.

## Data availability statement

The original contributions presented in the study are included in the article/Supplementary material, further inquiries can be directed to the corresponding author.

## Ethics statement

The present study was in accordance with ethical standards related to College of Languages and Translation, Preparatory Year Deanship, Imam Mohammad Ibn Saud Islamic University. Participants provided their informed consent to participate in this study.

## Author contributions

The author contributed directly to the conceptualization of the content, literature selection and review, and manuscript writing-up and revision. He contributed to the article and approved the submitted version.
