# Supplementary material for: Students' boredom in English language classes: Voices from Saudi Arabia
Source: Front Psychol. 2023 Mar 7;14:1108372. doi: 10.3389/fpsyg.2023.1108372 (PMC10029976; doi:10.3389/fpsyg.2023.1108372)
Supplement: Supplementary file 1 [file Data_Sheet_1.PDF]

Appendix (A)

Boredom Questionnaire

Demographic Information

Gender Male ( ) Female ( )

The type of Academic Program 1- Preparatory Year Deanship ( ) 2- College of Languages and Translation ( ) 3- Other Colleges ( )

Dear student

This questionnaire aims at exploring the reasons beyond boredom when attending English classes. Please note that this is not a test as there are no "right" or "wrong" answers to its questions. Therefore, please try to give as honest answers as possible. I confirm that the questionnaire data will be only used for research purposes and that your privacy will be confidential. Thank you in advance for your cooperation and for taking some time in completing the questionnaire.

**Classroom disengagement and monotony**	**Always**	**Often**	**Occasionally**	**Seldom**	**Never**
1. Time passes slowly in English classes.					
2. It takes me more time to get engaged in English classes than other students around me.					
3. I do not feel like doing anything in English classes.					
4. It is not easy for me to concentrate in English classes.					
5. During English classes, I often think about unrelated things.					
6. I just sit around doing nothing in English classes.					
7. I often find myself at loose ends in English classes.					
8. I do not feel entertained or excited in English classes.					

Do you have any other negative feelings in language classes? If so, please explain.

Task-related boredom

I feel bored in English classes when….	Always	Often	Occasionally	Seldom	Never
9. Learning activities are easy.					
10. Learning activities very difficult.					
11. Doing similar learning activities.					
12. Online learning activities.					
13. Face-to-face learning activities.					
14. Doing learning activities in my own.					
15. Doing group learning activities.					
16. Doing pair activities.					
17. Doing grammar activities.					
18. Doing listening activities.					
19. Doing speaking activities.					
20. Doing pronunciation activities.					
21. Doing writing activities.					
22. Doing vocabulary activities.					
23. Doing reading activities.					

Are there any other language learning activity types which make you feel bored in language classes? If so, please explain.

Teacher-related boredom

I feel bored in English classes when the teacher....	Always	Often	Occasionally	Seldom	Never		
24. Has unchanging instructional routines.							
25. Has excessive control over the class.							
26. Does not engage me in learning activities							
27. Overloads me with language information.							
28. Corrects my mistakes.							
29. Does not correct my mistakes.							
30. Is not friendly.							

Are there any other teacher-related factors which make you feel bored in language classes? If so, please explain.

Language learning ability-related boredom

I feel bored in English classes because ….	Always	Often	Occasionally	Seldom	Never
31. I have a limited English language ability.					
32. I cannot see any progress in my English language ability.					
33. I am not efficient in the language area covered in the learning activity.					
34. I feel my English level is much higher than my peers.					
35. I feel my English level is much lower than my peers.					

- Are there any other feelings which make you have bored in language classes? If so, please explain.
